# Rapid Loss of Tidal Flats in the Yangtze River Delta since 1974

**DOI:** 10.3390/ijerph17051636

**Published:** 2020-03-03

**Authors:** Xing Li, Xin Zhang, Chuanyin Qiu, Yuanqiang Duan, Shu’an Liu, Dan Chen, Lianpeng Zhang, Changming Zhu

**Affiliations:** 1 School of Geography, Geomatics and Planning, Jiangsu Normal University, Xuzhou 221116, China; qiucy2012@163.com (C.Q.); dyq@jsnu.edu.cn (Y.D.); liushuan@jsnu.edu.cn (S.L.); cdchen@jsnu.edu.cn (D.C.); zhanglp@jsnu.edu.cn (L.Z.); zhuchangming@jsnu.edu.cn (C.Z.); 2 State Key Laboratory of Remote Sensing Science, Institute of Remote Sensing and Digital Earth, Chinese Academy of Sciences, Beijing 100101, China

**Keywords:** Yangtze River Delta, tidal flat, remote sensing, Landsat, reclamation, sediment supply

## Abstract

As the home to national nature reserves and a Ramsar wetland, the tidal flats of the Yangtze River Delta are of great significance for ecological security, at both the local and global scales. However, a comprehensive understanding of the spatiotemporal conditions of the tidal flats in the Yangtze River Delta remains lacking. Here, we propose using remote sensing to obtain a detailed spatiotemporal profile of the tidal flats, using all available Landsat images from 1974 to 2018 with the help of the Google Earth Engine cloud platform. In addition, reclamation data were manually extracted from time series Landsat images for the same period. We found that approximately 40.0% (34.9–43.1%) of the tidal flats in the study area have been lost since 1980, the year in which the tidal flat area was maximal. The change in the tidal flat areas was consistent with the change in the riverine sediment supply. We also found that the cumulative reclamation areas totaled 816.6 km^2^ and 431.9 km^2^ in the Yangtze estuary zone and along the Jiangsu coast, respectively, between 1974 and 2018. Because of reclamation, some areas (e.g., the Hengsha eastern shoal and Pudong bank), which used to be quite rich, have lost most of their tidal flats. Currently, almost 70% of the remaining tidal flats are located in the shrinking branch (North Branch) and the two National Nature Reserves (Chongming Dongtan and Jiuduansha) in the Yangtze estuary zone. Consequently, the large-scale loss of tidal flats observed was primarily associated with reduced sediment supply and land reclamation at the time scale of the study. Because increasing demand for land and rising sea levels are expected in the future, immediate steps should be taken to prevent the further deterioration of this valuable ecosystem.

## 1. Introduction

Tidal flats are among the most important ecosystems in the world [1]. They play an irreplaceable role in water purification, carbon sequestration, biodiversity conservation, and storm surge protection [2,3]. Tidal flats are also highly vulnerable ecosystems and are particularly sensitive to global change [4]. Human activities and natural forces have led to a loss of approximately 25–50% of the world’s tidal flats [5,6], and it is predicted that they will continue to be lost at a substantial rate in the future decades [4]. Because tidal flats are alternately submerged and exposed to air by tides and consist of unconsolidated sediments, they are a highly dynamic environment, thereby providing limited access for researchers. Consequently, it is difficult for traditional measurement techniques to obtain sufficiently extensive data in tidal flats. Therefore, our knowledge concerning these ecosystems at the regional and local scales is insufficient to provide information for the development of practical countermeasures to address the regional and global loss of tidal flats [7].

Remote sensing techniques provide alternative approaches to mapping the areal extent of tidal flats. Using a water index image differencing procedure, Murray et al. [8] mapped the extent of the tidal flats across East Asia and along the Yellow Sea, using Landsat images at the continental scale. Instead of attributing tidal height to a single scene, as done by Murray et al. [7], Sagar et al. [9] developed an automated method to model the continental-scale intertidal extent by using all Landsat scenes and the OTPS TPX08 model, based on the Australian Geoscience Data Cube Coastal Cells (1° × 1°). Using a similar method to that of Murray et al. [7], Chen et al. [10] estimated tidal flat areas in the Yangtze River Delta using Landsat images from seven time periods, between 1985 and 2014. Sun et al. [11] classified coastal wetlands in the Yangtze River Delta, including tidal flats, using a decision tree method based on 7-year Landsat/GF/SPOT images between 1979 and 2016. These studies simplified spatial variations of the tidal level by applying a single tidal height to an entire scene or cell (1°×1°). As indicated by Sagar et al. [12], this simplification produces a significant error when applied at a large scale or to a complex study area. Thus, in an updated version [13], they used a Voronoi partitioning method to permit spatial variations of the tidal level. Such partitioning can provide a more accurate model of the extent of the tidal flat and, therefore, derive a more accurate tidal flat digital elevation model. These studies required the use of tidal models or tide level data to obtain knowledge of tides in the study area, thereby limiting the large-scale application of this technique [14].

**Figure 1:**
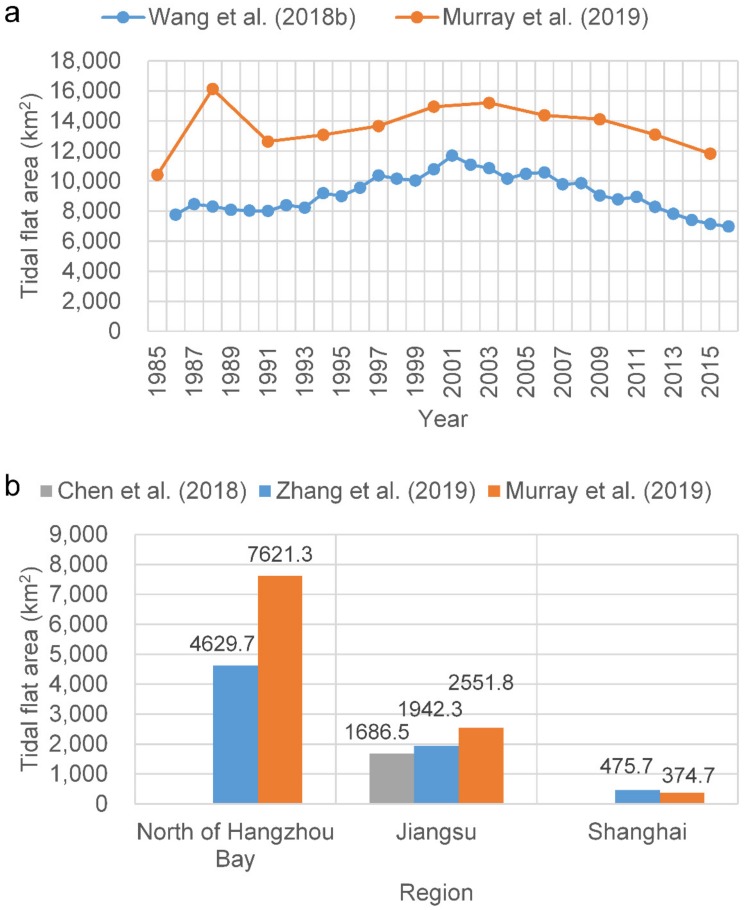
Comparison of the studies [14,15,17,18] regarding the extraction of tidal flats in the coastal zones of (**a**) China and (**b**) north of Hangzhou Bay, Jiangsu Province, and Shanghai for 2014–2016. In subregion b, the results of Zhang et al. [15] and Murray et al. [14] were from 2014–2016, whereas the result of Chen et al. [18] was from 2016.

Some recent studies have attempted to overcome this dependence on tidal information [14,15,16,17]. Murray et al. [14] used the random forest classification algorithm with 56 predictor variables within each three-year period in the Google Earth Engine (GEE) platform to map the global extent of and change in tidal flats between 1984 and 2016. Zhang et al. [15] developed a random forest algorithm via GEE to classify tidal flats in the China eastern coastal zone, circa 2015, using the statistics of six spectral indices and six spectral bands within a three-year timeframe. These two studies avoided the dependence on inputs concerning local tidal levels and, thus, can be applied at the national and global scales. Zhao et al. [16] used a quantile synthesis method for each pixel of time series Sentinel-1 Synthetic Aperture Radar (SAR) images to estimate the tidal datum, under which the tidal flat extent can be identified through the automated classification of land and water using the Otsu method. Wang et al. [17] employed all available Landsat images in the GEE platform and a decision tree algorithm to generate maps of annual tidal flats, using annual frequency maps of an open surface water body and vegetation. The water frequency method does not require knowledge of tidal elevations at the time of image acquisition; thus, it is, theoretically, an ideal globally applicable method. However, it is difficult to obtain ideal results in actual applications, particularly in coastal environments, where highly turbid water is prevalent, including in the Yangtze Delta. The main reasons for this include the following: (1) it is difficult to accurately determine the position of the waterline by thresholding in highly turbid water across a large study area; (2) thresholding the water frequency tends to exclude data derived from images taken during periods of lower water level, thus causing significant inaccuracies in resulting estimates of the tidal flat extent. The differences in these studies are mainly in the data and methodology. Only Zhao et al. [16] used the SAR images, whereas the others used all available Landsat images during specific time periods. With respect to methodology, both Murray et al. [14] and Zhang et al. [15] used the random forest algorithm to directly derive the tidal flat during specific time periods (e.g., 2014–2016); however, the difference between the two studies is that different variables were employed for the algorithm. Both Wang et al. [17] and Zhao et al. [16] combined multiple methods, and essentially, the quantile synthesis method of Zhao et al. [16] and the annual frequency mapping of Wang et al. [17] have something in common—both used the threshold method. Consequently, the results from the published studies differed widely at the regional scale (Figure 1). For example, Wang et al. [17] determined that the area of tidal flats in China was 7311.7 km^2^ in 2016; however, Murray et al. [14] estimated an area of 12,049 km^2^ (65% higher), between 2014 and 2016. Zhang et al. [15] determined that the tidal flat areas in Jiangsu Province and Shanghai were 1942.3 km^2^ and 475.7 km^2^, respectively, between 2014 and 2016, whereas Chen et al. [18] estimated the area for Jiangsu Province to be 1686.45 km^2^ (13% lower) in 2016, and Murray et al. [14] estimated the tidal flat areas in Jiangsu Province and Shanghai to be 2551.8 km^2^ and 374.7 km^2^, 31% higher and 21% lower than the estimations of Zhang et al. [15], respectively. Zhang et al. [15] also estimated that the tidal flat area was 4629.7 km^2^ along China’s eastern coast from the Yalu River to Hangzhou Bay, while Murray et al. [14] estimated the area to be 7621.3 km^2^ (65% higher). Thus, the accurate derivation of the extent of tidal flats at the regional scale still faces great challenges.

According to Semeniuk [19], the tidal flat is located between the lowest tide level at the equinoctial low-water spring tide and the highest tide level at the equinoctial high-water spring tide, and the concept of a tidal flat sometimes varies with authors and between disciplines. As for the extraction of the tidal flat extent from satellite images, field survey data or higher-resolution image data are very important for validating the results. However, in reality, owing to the constantly rising and falling of tides and the poor accessibility of the tidal flat, it is almost impossible to obtain a “real”, large-scale and long-term tidal flat extent by field surveys or high-resolution imaging. Therefore, one of the main purposes of our study is to develop a remote sensing approach that can acquire tidal flat extent data that are as real as possible, through satellite images. We determined the extent of the tidal flats in the Yangtze River Delta for each year between 1974 and 2018. We also explored the impact of influencing factors, including reclamation, estuarine projects, sediment supply, and relative sea-level rise, which may affect the spatiotemporal dynamics of the tidal flats. Ultimately, this study produces a detailed picture of the spatiotemporal changes in the tidal flats in the Yangtze River Delta over the past 45 years. This work will serve as a reference for the sustainable management of tidal wetlands. The study area includes the Jiangsu coast, south of Xiaoyangkou, and the Yangtze estuary north of the Nanhui spit in Shanghai, an area covered by a single Landsat scene.

## 2. Materials and Methods

**Figure 2:**
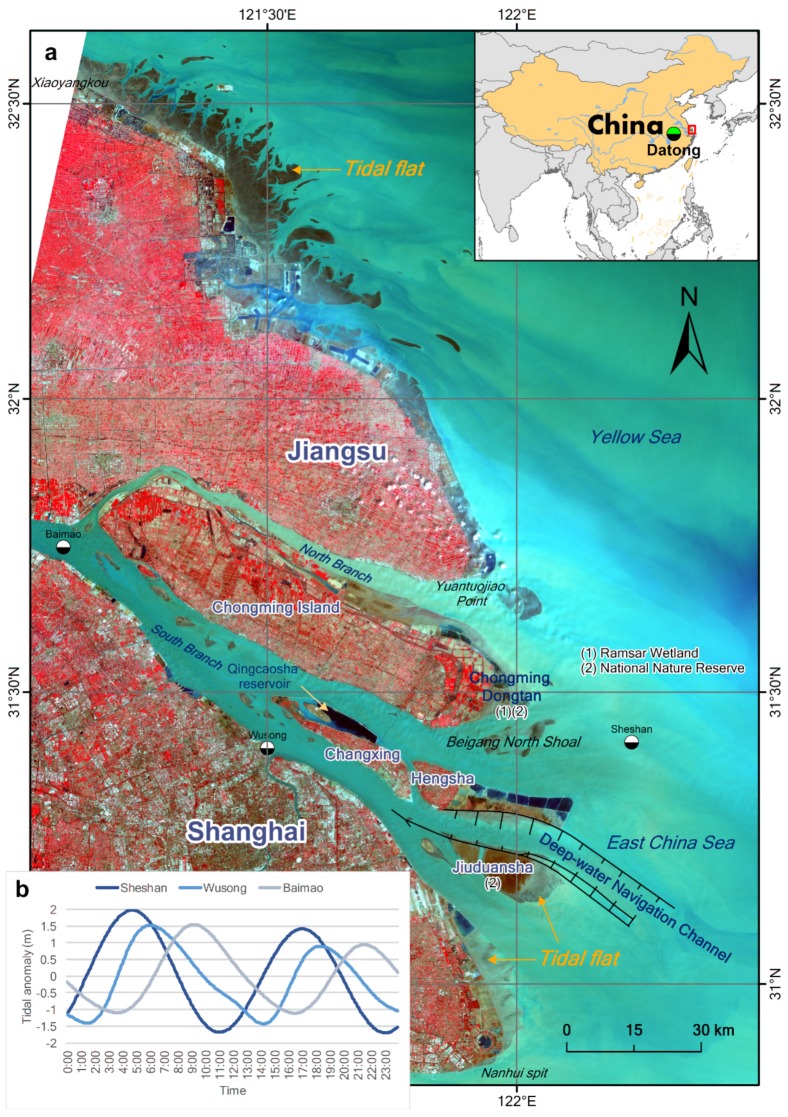
(**a**) Geographical settings of the Yangtze River Delta. The underlying image is based on the Landsat 8 OLI data acquired on April 2, 2017, with a 5–4–3 band combination. Vegetation is displayed in red and water in blue. (**b**) Tidal heights are relative to the mean sea level from the International Hydrographic Organization tidal database over a one-day period on January 1, 2017 at the Sheshan, Wusong, and Baimao stations. The Sheshan station is approximately 25 km east of Chongming Dongtan. The Wusong station is in the Huangpu River estuary. The Baimao station is near the bifurcation of the southern and northern branches. The trends in tidal level changes at the Sheshan and Baimao stations are almost opposite, which reflects the large spatial variations in the tidal level in the study area.

The tidal flats of the Yangtze River Delta (Figure 2) are some of the most important estuarine wetlands in China. Chongming Dongtan is a Bird National Nature Reserve and a Ramsar Wetland of International Importance, whereas Jiuduansha is a Wetland National Nature Reserve. Both wetlands are located in the central part of the East Asian–Australian flightpath for migratory birds. Thus, the tidal flats of the Yangtze River Delta play a vital role in local and global biodiversity conservation. However, the local government of the Yangtze River Delta, representing the world’s sixth-largest metropolitan area [20], used tideland reclamation as the primary means by which to meet the increasing demand for land, which is driven by rapid economic and urban growth in the study area. At present, some of the large-scale reclamation projects in the Yangtze estuary zone include the Eastern Hengsha and Pudong coastal beach reclamations. In addition, there will be large-scale reclamation along the Jiangsu coast, according to the “Outline of Jiangsu Coastal Reclamation Development Plan” [21]. Therefore, it is very important to determine the inventory and distribution of tidal flats in the Yangtze River Delta to maintain the balance between development and protection. Unfortunately, comprehensive knowledge of the status of tidal flats in the Yangtze River Delta remains largely unknown, despite several published studies.

Landsat images have been successfully used to obtain information regarding tidal flats in many parts of the world (e.g., [7,9,10,22,23,24]). To retrieve the annual extent of the tidal flats in the Yangtze Delta, from 1974 to 2018, we used Landsat images as the data source. The entire Landsat data archive is now freely available, courtesy of the US Geological Survey (https://earthexplorer.usgs.gov/) and the GEE data archive (http://earthengine.google.com). The Landsat multispectral scanner (MSS) images from GEE are Level-1 Precision Terrain (L1TP) processed data and the Landsat Thematic Mapper (TM)/ Enhanced Thematic Mapper (ETM+)/ Operational Land Imager (OLI) dataset has been processed with the orthorectified and atmospherically corrected surface reflectance. In addition, we used the sediment discharge data from Datong station to explore the influence of changes in sediment discharge on the extent of the tidal flats. The sediment discharge data are available from the Changjiang Sediment Bulletin published by the Changjiang Water Resources Commission (http://www.cjw.gov.cn/). Datong station is the gauging station nearest the estuary and tidal limit of the Yangtze River (Figure 2).

**Figure 3:**
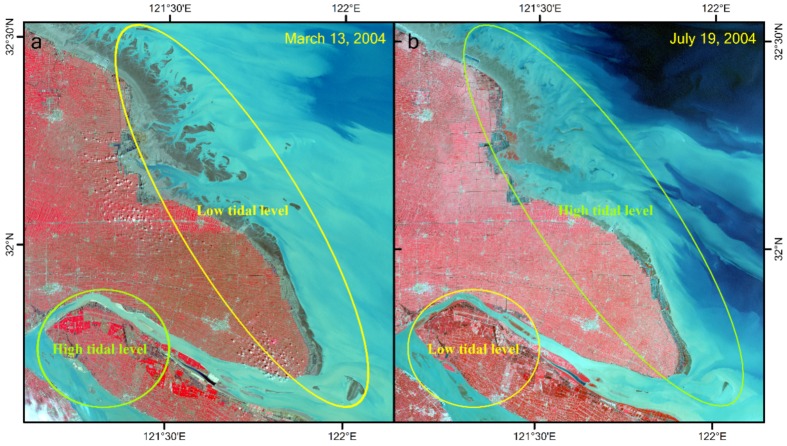
Sample images demonstrating large spatial variations in the tidal level in the study area. (**a**) The Jiangsu coast is at a low tidal level, whereas the upper estuary is at a high tidal level in the image from March 13, 2004; (**b**) this is reversed in the image from July 19, 2004.

The study area has two unfavorable traits for accurate extraction of the extent of tidal flats. First, the tidal level varies significantly along its approximately 1000 km coastline (Figure 2 and Figure 3), although the study area can be covered by only a single Landsat image scene (WRS-2 path 118, row 38). Consequently, significant errors are unavoidable when extracting the areal extent of the tidal flats within a single scene if the spatial variations in tidal levels are not considered. Second, the study area is located in a large estuary with optically complex water conditions. In particular, the highly turbid coastal waters invalidate many of the tidal flat extraction methods, including the median compositing method [9], water frequency method [17], and some spectral index methods (e.g., normalized difference water index (NDWI) and modified normalized difference water index (MNDWI)).

Because of the relatively long revisit intervals (16 or 18 days) of the Landsat satellites, and frequent cloud coverage in the Yangtze Delta region, the availability of Landsat images are very limited. The key to obtaining the real extent of tidal flats is to accurately acquire the waterline at the lowest possible tidal level in the whole study area within a specified period of time. However, owing to the large spatial variation in the tidal level in the study area, the occurrence of the low tide in some areas of the study area coincides with the occurrence of the high tide in other areas within a single image scene (Figure 3). Consequently, if a single scene with a low tide at one location was used to extract the extent of the entire tidal flat, the results would inevitably deviate significantly from the true value. An ideal scenario would be that the derived tidal flats are at the lowest possible tide everywhere in the study area at a specified time window. An approximate method is to partition the study area, based on tidal information. However, the patterns in the spatial variation of tides do not remain constant over time; therefore, it remains a challenge to explore the long-term changes of the extent of tidal flats from satellite images at a large spatial scale.

A lower tidal level corresponds to a larger areal extent of a tidal flat. Therefore, we can estimate the extent of a tidal flat as accurately as possible by considering the union of all extraction results of tidal flat extents from all available Landsat scenes in a specified year. The union is the maximal extent of the tidal flats, comprising all the extents of the tidal flats in a year. The key step in implementing this method is to accurately depict the waterline or classify the tidal flat. The simplest method is a single or combined spectral indices method, such as NDWI or MNDWI, both of which have been widely used to extract tidal flats [8,9,24]. Wang et al. [17] used the expression “EVI < 0.1 and (MNDWI > EVI or MNDWI > NDVI)” to identify open water. EVI represents the enhanced vegetation index [25]. We found that these methods are very effective in clear water; however, they do not work well in turbid water with high sediment concentrations, such as the Yangtze River estuary (see Section 4.3). This is one of the main reasons for the large differences in published research results.

In light of these problems, we decided to use a classification method to extract the extent of the tidal flats in the Yangtze Delta. First, we created feature images by combining the original spectral bands and spectral indices of all available images. Second, we selected some original bands and spectral indices, such as the normalized difference vegetation index (NDVI), NDWI, and MNDWI, for further analyses. We stacked these bands and spectral indices into new images as the data source for our supervised classifier. Third, we used the classification and regression tree (CART) classifier to assign pixels to one of two classes: “open water” or “land.” The “land” class included tidal flat and other land covers on land. Finally, to implement the CART model, we selected different training samples for each available Landsat scene by referring to the high-resolution Google Earth image and time-series Landsat archives. The GEE cloud platform was used to conduct the classification, allowing us to check the classification results in real-time. Thus, it was simple and convenient for us to modify the training samples until we obtained a satisfactory classification result. We determined the lower boundary of the tidal flat by taking the union of the classification results of all the available Landsat images in a year.

The NDVI is one of the most commonly used spectral indices and has been successfully utilized to discern the vegetation on tidal flats [26]. The NDVI was also able to effectively extract the waterline on a tidal flat because it can enhance the difference between the exposed tidal flat and turbid water [27]. The NDWI [28] and MNDWI [29] are two of the most effective remote sensing indices for identifying water bodies and have been widely applied in recent decades [30]. To aid in the more accurate extraction of the tidal flat, we also constructed two new spectral indices: normalized difference tidal flat index (NDTI) and turbid water index (TWI). These indices were calculated from the following:

(1) $$\mathrm{NDTI}=\frac{b_{red}-b_{nir}-b_{swir}}{b_{red}+b_{nir}+b_{swir}}$$

(2) $$\mathrm{TWI}=\frac{b_{nir}-5b_{swir}}{b_{nir}+5b_{swir}}$$

where subscripts red, nir, and swir represent red, near-infrared, and short-wave infrared, respectively. For Landsat 8 OLI images, *b_red_*, *b_nir_*, and *b_swir_* correspond to bands 4, 5, and 7, respectively, and for Landsat TM or ETM+ images, *b_red_*, *b_nir_*, and *b_swir_* correspond to bands 3, 4, and 7, respectively. The NDTI has similar characteristics to the MNDWI but exhibits an enhanced capacity to distinguish between high moisture tidal flats and turbid water, compared with that of the MNDWI. The NDTI is relatively insensitive to the impact of cloud cover. In TWI images, the bright tone of turbid water contrasts against the dark tone of clear water; such, the TWI can differentiate between clear and turbid waters.

For Landsat 8 images, we selected the six original bands, b3 to b7, b10, and four spectral indices, NDVI, MNDWI, NDTI, and TWI; for Landsat 5 and 7 images, we selected the six original bands, b2 to b7, and four spectral indices, NDVI, MNDWI, NDTI, and TWI; and for Landsat 1-3 MSS images, we selected the four original bands, b1-b4, and two spectral indices, NDVI and NDWI.

Vegetation zones occur along most of the coast in the study area; therefore, the vegetation line can be employed as a proxy of the high tide waterline, and for some excessively reclaimed coasts where vegetated areas are rare outside of the reclamation dike, the dike itself can be used as a proxy for the high tide waterline [31]. The position and timing of the reclamation dikes are easy to manually identify from Landsat time-series images. Because the vegetation line varies with the seasons, we first generalized all NDVI images of all available Landsat images in a year into an annual maximum NDVI image. Then, we thresholded the annual maximum NDVI image to obtain a “vegetation line” as the final upper boundary of the tidal flat. In this way, we depicted the extent of the tidal flat by defining the upper and lower boundaries. Some temporary tidal flats were not considered in our study. These tidal flats formed during the reclamation process, e.g., those in the Hengsha eastern shoal and Pudong bank, and will be converted into other land use types over a short period of time.

**Figure 4:**
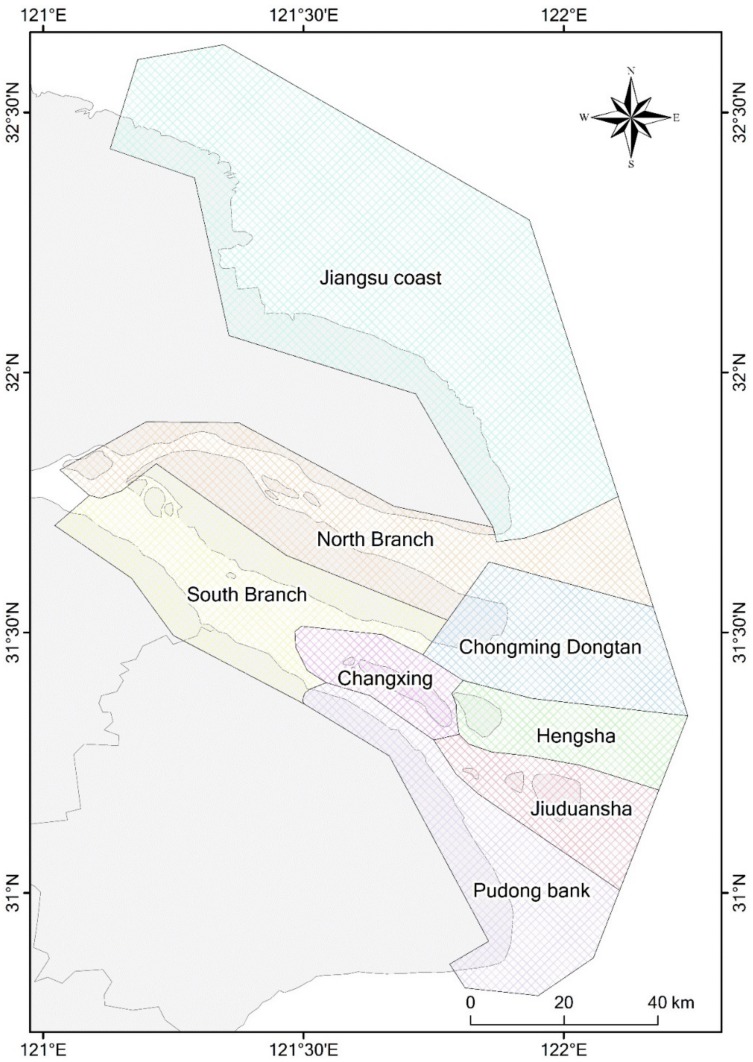
Map of the sub-divisions of the study area.

To facilitate detailed spatial analysis, we divided the study area into the Jiangsu coast and Yangtze estuary zone, by Yuantuojiao Point. The Yangtze estuary zone was then further divided into seven sub-regions, namely, the North Branch, the South Branch, Chongming Dongtan, Hengsha, Changxing, Jiuduansha, and Pudong bank (Figure 4). Different sub-regions have different physical settings, in which the tidal flats could behave differently. For example, the North Branch is a shrinking branch; the South Branch is an inner-channel environment; Chongming Dongtan and Jiuduansha are national nature reserves, and: Hengsha and Pudong banks are undergoing large-scale reclamation. Thus, our detailed spatial analysis provides a comprehensive understanding of the tidal flats in the study area. Furthermore, it can offer possible inspiration for studying the evolution of the tidal flats in different physical settings.

## 3. Results

### 3.1. Overall Spatiotemporal Dynamics

**Figure 5:**
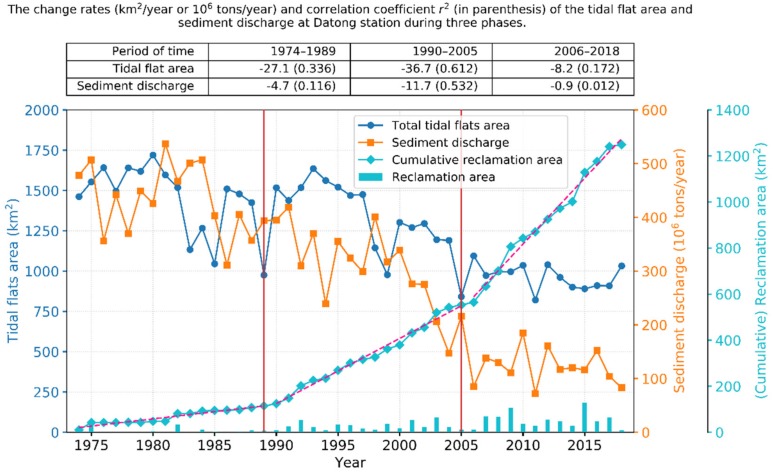
Temporal changes in the total tidal flat area, cumulative reclamation area in the study area, and sediment discharge at the Datong station since 1974. The tidal flat area exhibited a general decreasing trend that remained approximately consistent with that of the sediment discharge. Reclamation activities in the study area can be clearly divided into three phases by the years 1989 and 2005, during which reclamation gradually intensified. The upper table shows the change rates of the tidal flat area and sediment load at Datong station and their corresponding correlation coefficients *r*^2^ (in parenthesis) during the three phases.

In general, the total areal extent of the tidal flats in the study area has declined significantly (*r*^2^ = 0.622) since 1974 but was maximal in 1980 (Figure 5). The changes were generally consistent with changes in the riverine sediment load (*r*^2^ = 0.489), whereas the reclamation had a significant influence on the loss of tidal flats. The Jiangsu coast and Yangtze estuary zone reached a maximal tidal flat area of 1719.8 km^2^ and 548.8 km^2^, respectively, in 1980. Between 1980 and 2018, 40.6% and 38.8% of the tidal flats in these areas were lost, equating to an average loss of 12.5 km^2^/year and 5.6 km^2^/year, respectively. Most of the tidal flats were distributed along the Jiangsu coast during the past 40 years. In 1980, tidal flats in the Yangtze estuary zone were mainly located in the four sub-regions, Chongming Dongtan, the North Branch, Pudong bank, and Jiuduansha, which together constituted 78.7% of the total area of the tidal flats in the estuary zone. At present, the tidal flats along the Pudong bank have been largely lost because of reclamation. The tidal flats in the Yangtze estuary zone are mainly located in Chongming Dongtan, the North Branch, the South Branch, and Jiuduansha, which together occupy 74.3% of the total area of the tidal flats in the estuary zone.

Reclamation in the study area can be clearly divided into three phases, delineated by the years 1989 and 2005, during which reclamation gradually intensified and the tidal flats exhibited different patterns of change (Figure 5).

Between 1974 and 1989, the tidal flat area exhibited an overall small decline (*r*^2^ = 0.336). The tidal flat area increased prior to 1980 and decreased with large fluctuations between 1980 and 1989, during which the tidal flat area reached the smallest area (975.3 km^2^) in 1989 and the second smallest value was recorded in 1985. The tidal flat areas in the Yangtze estuary zone and along the Jiangsu coast declined by 22.0% and 37.5%, respectively, from 1974 to 1989; however, both were reduced by up to 43.3% from 1980 to 1989. The corresponding sediment load at Datong station declined by 17.6% between 1974 and 1989. There was limited reclamation prior to the 1980s, after which reclamation began to gradually increase. Between 1974 and 1989, 81.3 km^2^ and 32.9 km^2^ of coastal land were lost because of reclamation along the Jiangsu coast and in the Yangtze estuary zone, respectively.

Between 1990 and 2005, the total tidal flat area significantly declined (*r*^2^ = 0.612). By 2005, the tidal flat areas in the Yangtze estuary zone and along the Jiangsu coast declined to 194.8 km^2^ and 646.5 km^2^, respectively, equivalent to reductions of 50.8% and 42.4%, respectively, from the 1990 level. Sediment load at Datong station also decreased by 45.3% during this period. The cumulative reclamation area in the Yangtze estuary zone reached 112.0 km^2^ from 1974 to 1992, overtaking that of the Jiangsu coast, for the first time. Between 1990 and 2005, 348.7 km^2^ and 90.2 km^2^ of coastal land were reclaimed in the Yangtze estuary zone and along the Jiangsu coast, respectively.

Between 2006 and 2018, the total tidal flat area remained approximately stable (*r*^2^ = 0.172) with an average of 966.0 km^2^. Similarly, sediment load remained largely unchanged during this phase. The tidal flat area along the Jiangsu coast decreased by 15.8%, whereas it increased in the Yangtze estuary zone by 25.4%, despite the prevalence of large-scale reclamation during this period. The total reclamation areas were 435.1 km^2^ and 260.5 km^2^ in the Yangtze and Jiangsu areas, respectively, from 2006 to 2018. The increase in the tidal flat area in the Yangtze estuary originates mainly from Chongming Dongtan and the South Branch (see Section 3.2). For Chongming Dongtan, the increase may be related to the shrinkage of the North Branch and the accretion of the Beigang North Shoal (Figure 2). For the South Branch, the cause is likely large-scale estuarine projects and reclamation inducing the variation in the hydrodynamic environment in the South Branch [32,33,34].

### 3.2. Sub-Regional Spatiotemporal Dynamics

**Figure 6:**
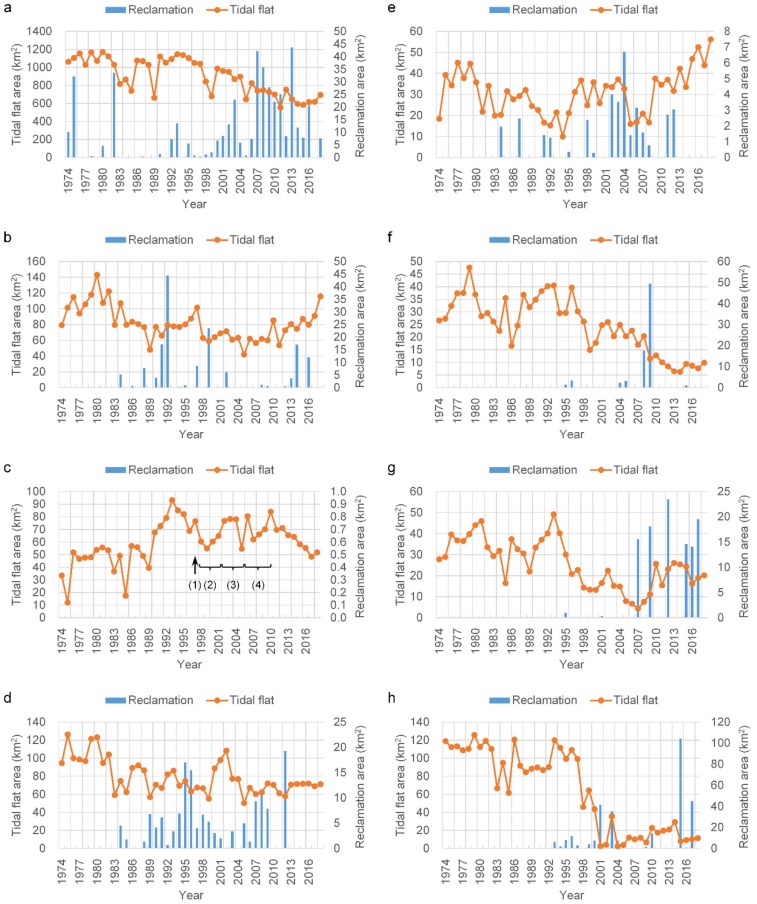
Changes in total areal extent of the tidal flats in the sub-regions of the study area from 1974 to 2018. (**a**) Jiangsu, (**b**) Chongming Dongtan, (**c**) Jiuduansha, (**d**) North Branch, (**e**) South Branch, (**f**) Changxing, (**g**) Hengsha, (**h**) Pudong. In subfigure c, (1) *S*. *alterniflora* was introduced in Jiuduansha for the purpose of ecological engineering; (2) During 1998–2001, the first phase of the Deep Water Navigation Channel (DNC) project was implemented; (3) During 2002–2005, the second phase of the DNC was undertaken; (4) During 2006–2010, the third phase was carried out.

As shown in Figure 6a, prior to 1993, along the Jiangsu coast, the area of tidal flats remained approximately unchanged (*r*^2^ = 0.004), with some fluctuations and the mean area was 1030.2 km^2^. Subsequently, these tidal flats showed a significant decline (*r*^2^ = 0.754) with a 39.4% reduction by 2018, equal to an average annual loss of 18.1 km^2^ of coastal land. Land reclamation for ports, salt ponds, and aquaculture development were the main factors that caused the loss of tidal flats. Intermittent reclamation occurred throughout the 20 years from 1974 to 1994, with a cumulative area of 103.6 km^2^ reclaimed. Because of the implementation of the “marine Sudong” development strategy, reclamation activities have occurred nearly every year since 1995. In 2009, the Development Plan of the Coastline of Jiangsu Province indicated that coastal development planning was officially the state strategy of China. Therefore, new reclamation planning will likely lead to additional loss of tidal flats.

Chongming Dongtan has been a primary research focus in the past because it is a National Nature Reserve and also a Ramsar Wetland of International Importance. As shown in Figure 6b, the tidal flat area decreased (*r*^2^ = 0.489) between 1974 and 2005, with a maximum of 142.9 km^2^ in 1980 and a minimum of 41.9 km^2^ in 2005. Between 2005 and 2018, the tidal flats significantly increased (*r*^2^ = 0.721) in areal extent, to a total of 115.5 km^2^ in 2018, approximately three times that of 2005, and close to the area observed around 1980. Remote sensing observations have indicated that reclamation activities have been ongoing since 1984, and the cumulative reclaimed area reached 153.6 km^2^ in 2018.

The Jiuduansha was designated as a Wetland National Nature Reserve in 2005. It is currently the only natural wetland with limited human interference in the Yangtze estuary. As shown in Figure 6c, the areal extent of the Reserve initially increased and then declined between 1974 and 2018. Its maximal area of 93.1 km^2^ was attained in 1993, with the second maximum of 84.1 km^2^ in 2010. After 2010, its area started to significantly decrease (*r*^2^ = 0.910) at a rate of 3.9 km^2^/year. In 1999 and 2005, the tidal flat areas had local minima of 54.9 km^2^ and 54.6 km^2^, respectively. Between 1998 and 2001, the Deep Water Navigation Channel (DNC) project implemented the first phase of the project, and in 2005, the second phase was completed; these phases were likely related to the two minima identified. In addition, Landsat images show that the existence of vegetation began the stable colonization of the Jiuduansha in 1990. Since 1997, when *S. alterniflora* was introduced for the purpose of ecological engineering, the vegetated area in Jiuduansha increased [35], leading to the stabilization of the formerly submerged shoals. Consequently, the tidal flat area declined because vegetation encroachment was more rapid than the growth of the tidal flat [35]. Meanwhile, with the completion of the DNC project in 2010, the combined influence is most likely the reason for the rapid decline of the tidal flat area since 2010.

As shown in Figure 6d, in the North Branch, the tidal flat area showed a decline (*r*^2^ = 0.393) in area between 1974 and 1989, from a maximum of 126.2 km^2^ in 1975 to a minimum of 56.9 km^2^ in 1989. Since 1990, the tidal flat area has remained stable (*r*^2^ = 0.004) with some fluctuations, with an average area of 71.7 km^2^. Reclamation activities started in 1984 and with increasing reclamation intensity, large areas of the tidal flats have been encroached upon along both banks of the North Branch. Thus, the tidal flat area gradually stabilized, with a cumulative reclamation area totaling 147.6 km^2^ since 1984.

In the South Branch between the first and second bifurcations, the tidal flats were mainly confined to two parts—first along the south bank and as shoals in the channel. Reclamation projects were also mainly located along the south bank, with lower intensity than those of other zones. The total reclamation area was 37.0 km^2^ between 1974 and 2018. As shown in Figure 6e, the areal extent of the tidal flat decreased (*r*^2^ = 0.415) before 1994 and then increased (*r*^2^ = 0.313) afterwards. After 2005, the increasing trend was particularly significant (*r*^2^ = 0.832). The early decline may have been related to reclamation activities; however, the subsequent increase was largely because of the growth of the shoals in the channel, which may have been associated with the changing hydrodynamic regime induced by the large-scale reclamation and navigation efforts in the Yangtze estuary [34].

As shown in Figure 6f, tidal flats at the perimeter of Changxing Island are overall degraded (*r*^2^ = 0.591). The largest area was 47.6 km^2^, occurring in 1979, and decreased to less than 10 km^2^ after 2011. Before the 1990s, the tidal flats appeared in most areas along the coast of Changxing Island. In particular, prior to 2000, there was a large area of natural tidal flats at the west end of Changxing Island. These tidal flats along the coast gradually became land over time owing to gradual accretion, eventually becoming vegetated. In 2008, 16.0 km^2^ of the tidal flats were converted to land through reclamation, and a tidal flat area of 50.6 km^2^ northwest of the Changxing Island was used for the construction of the Qingcaosha reservoir.

The tidal flats in Hengsha Island are located mainly on the Hengsha eastern shoal, and their recent areal evolution can be divided into two stages. As shown in Figure 6g, during the first stage, 1974–2007, the areal extent declined (*r*^2^ = 0.500), reaching a minimum of 4.4 km^2^ by 2007. Subsequently, the area gradually increased and became stable, with an average of 21.7 km^2^ after 2010. Reclamation in the Hengsha eastern shoal, containing a total area of 105.4 km^2^, began in 2003 and caused the early decrease in the tidal flat area. The original tidal flats on the eastern shoal were almost completely eliminated. The north training jetty and groins of the DNC project were completed in 2005 along the south side experiencing a tidal flat after 2008, which gradually grew to 18.3 km^2^ by 2018. Thus, the recent growth of tidal flats in Hengsha Island has been primarily influenced by navigation projects, however, this growth is limited because of its location in the navigation channel. Unrestricted expansion would negatively affect the ease of navigation and, thus, is not allowed.

As shown in Figure 6h, the change in the tidal flat area along the Pudong bank can be divided into two stages, before and after 1997. Between 1974 and 1997, the tidal flat area largely remained stable, with an average area of 101.8 km^2^, despite some fluctuations with time. Since 1998, the area of the tidal flats declined considerably, exhibiting a mean area of only 18.7 km^2^. This decline was mainly attributable to reclamation. Large-scale reclamation began in 1993, and the cumulative reclamation area reached 293.4 km^2^ in 2017. Large reclamation projects for the Pudong International Airport expansion began in 2008, with a reclamation area of approximately 164 km^2^. As a result, the South Passage was narrowed by nearly one-third, which will probably exert a significant influence on the hydrodynamic regime of the estuary [32].

## 4. Discussion

### 4.1. Impacts of Estuarine Projects and Sediment Supply

Shanghai is the largest megacity in the Yangtze River Delta and in China. Urban development activities in Shanghai have a major influence on tidal flats. Approximately 65% of Shanghai’s land was built on tidal flats that resulted from the accumulation of riverine sediment over the past 6000 years. By reclamation of these tidal flats, Shanghai city created more than 1200 km^2^ of new land over the past 60 years [36]. Land reclamation was and will remain the main means by which the land shortage in Shanghai is alleviated. The rate of urban land expansion is increasingly related to GDP growth [37]. According to the Shanghai Municipal Statistical Bureau [38], Shanghai’s population has grown from 11.0 million in 1978 to 24.2 million in 2017, and Shanghai’s GDP has risen from 27.3 billion Yuan in 1978 to 3063.3 billion Yuan in 2017. The urbanized area in Shanghai has increased from 12% in 1995 to 32% in 2015 [39]. The 13th Five Year Plan of Shanghai proposed that Shanghai will become an international economic, finance, trade, and shipping center by 2020. In the context of a reduction in the supply of riverine sediment, the conflict between land reclamation and tidal flat protection will further intensify as urban development continues.

Our results indicate that reclamation and reduced sediment load are the two main causes of the loss of tidal flats in the study area. The correlation coefficient (*r*^2^) between sediment load at Datong station and total tidal flat area in the entire study area (0.489), was larger than that (0.457) between sediment load and the tidal flat area in the Yangtze estuary zone. This implies that there is a sediment exchange between the Yangtze estuary and the Jiangsu coast, in accordance with the study of Wu and Wu [40]. The resilience of the tidal flats to reclamation activities is related to the sediment supply and accretion rate. This resilience will be improved when the sediment supply and accretion rate are sufficiently large. Thus, the impacts of reclamation are spatially dependent on the differences in accretion rates in different tidal flats. When the reclamation intensity does not exceed the threshold of the accretion rate, tidal flats may recover and shift seaward, even over a short period of time. Chongming Dongtan is an area where tidal flats have shown a net growth rate of 3.8 km^2^/year, despite a cumulative reclamation area of 35.0 km^2^ between 2005 and 2018 (Figure 6b). In contrast, Murray et al. [7] did not observe this phenomenon along the coast of the Yellow Sea, probably because lower spatial resolution images (250 m resolution) were used. If the reclamation intensity exceeds the threshold, tidal flats may need a longer time to recover and may even be unrecoverable. Both the Hengsha eastern shoal (Figure 6g) and Pudong bank (Figure 6h) have this characteristic. The Pudong bank was once among the main site of sediment deposition from the Yangtze River; however, these tidal flats have nearly disappeared owing to large-scale reclamation since 2000.

Large-scale reclamation and navigation projects, including reclamation along the Hengsha eastern shoal and Pudong bank and the DNC project, have also greatly modified the hydrodynamic regime in the Yangtze estuary [32,33,34], which may have led to the variations observed in the histories of these tidal flats.

### 4.2. Impacts of Relative Sea-Level Rise

According to the China Sea Level Bulletin 2018, released by the State Ocean Administration of China, sea levels along the Yangtze River Delta will rise 75–155 mm during the next 30 years. The rate of sea-level rise in the Yangtze estuary is estimated to be 5.44 mm/year, nearly triple the global average [41]. The study area is also within one of the most serious land subsidence areas in China [42], with a land subsidence rate of greater than 25 mm/year in 2014 and cumulative subsidence of 200 mm between 1985 and 2014 in most regions along the Jiangsu coast of the study area [43]. In Shanghai, the maximum land subsidence rate reached 24.12 mm/year [44], whereas the average subsidence rate decreased to 5.5 mm/year during the period of 2001–2011 [45]. The land subsidence will further augment the relative sea-level rise in the study area.

In contrast to estuarine projects and reduced sediment supply, sea-level rise is a long-term and complex process affecting tidal flats. Sea-level rise may induce a rise in tidal level [46], trigger higher storm surges [44], and alter the hydrodynamics [47] in the Yangtze estuary. These consequences of sea-level rise will inevitably lead to additional pressure on the fragile tidal flat ecosystems in the study area. Model simulations demonstrated that the combined effect of sea-level rise, land subsidence, and bathymetric change in the Yangtze estuary zone will cause an increase in coastal flooding by a factor of 8.5–23.4 by 2050 [48]. A new study has demonstrated that the study area is substantially more vulnerable to sea-level rise and coastal flooding than previous projections have suggested [49]. Therefore, in the impending climate change scenario, tidal flats and delta communities will be highly susceptible to sea-level rise and land subsidence, which will be further exacerbated by reduced sediment supply and increased human activity.

### 4.3. Accuracy Assessment and Uncertainty Analysis

**Figure 7:**
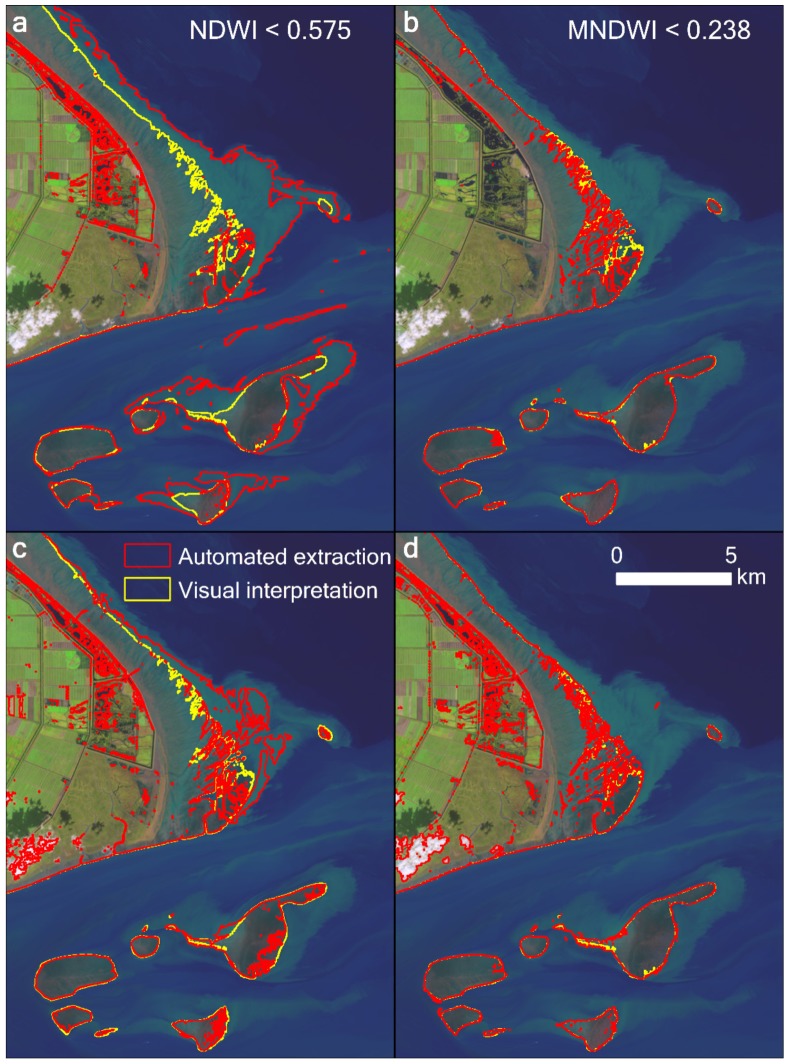
Comparison of the extracted tidal flat results using (**a**) NDWI, (**b**) MNDWI, and (**c**) the expression “EVI < 0.1 and (MNDWI > EVI or MNDWI > NDVI)” adopted by Wang et al. [17], and (**d**) our results in the highly turbid water body at Chongming Dongtan in the Yangtze estuary. The Landsat 8 image from October 30, 2018, was used, displayed with a 6–5–4 band combination.

**Figure 8:**
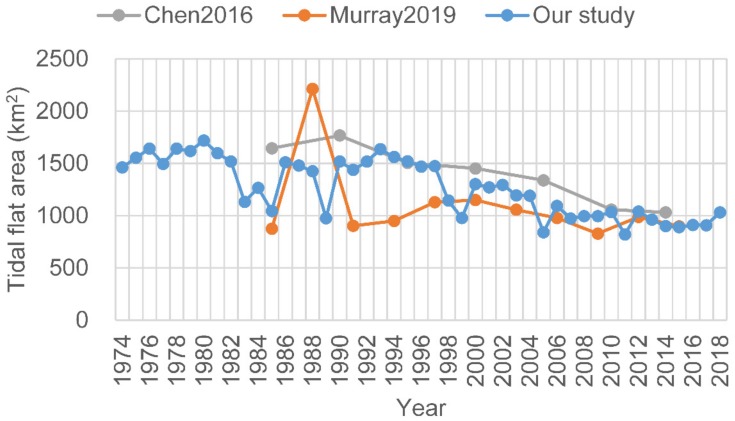
Comparison of the extracted results by Chen et al. [10] and Murray et al. [14] and our results. Good consistency was exhibited after 2000.

**Figure 9:**
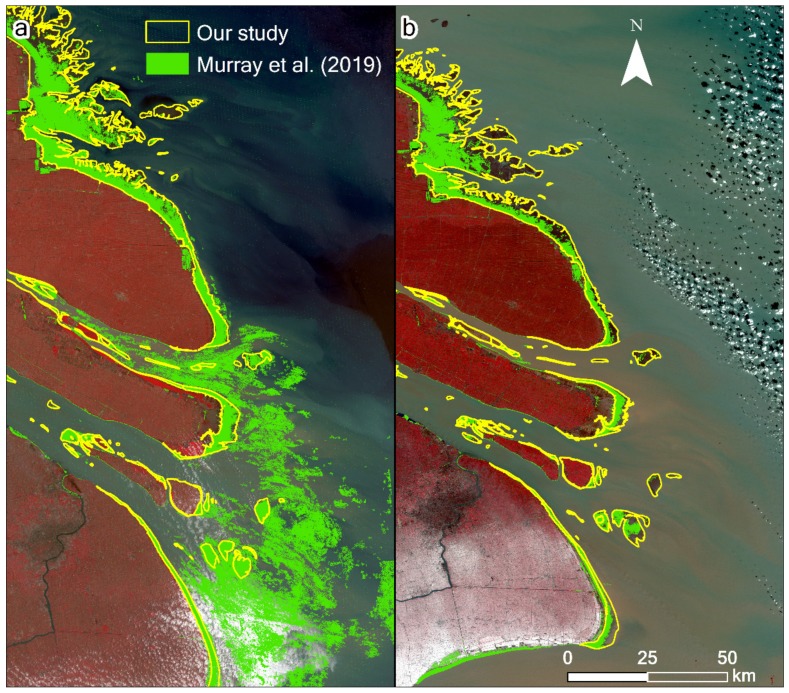
Comparison of the results of Murray et al. [14] and our results for the Yangtze River Delta in (**a**) 1988 and (**b**) 1991. The underlying images are based on the Landsat 5 TM data acquired on (a) July 5, 1988, and (b) January 5, 1991, respectively, with a 5–4–3 band combination. In the results of Murray et al. [14], some water bodies were clearly misclassified as tidal flats between 1987 and 1989, and some tidal flat pixels were omitted between 1990 and 1992.

In comparison, our results show better accuracy than those derived from NDWI, MNDWI, or the method adopted from Wang et al. [17] for the highly turbid water body of the study area (Figure 7). The MNDWI also performs well; however, there were some omissions near the easternmost tip of Chongming Dongtan. Our results are also consistent with those of Chen et al. [10] and Murray et al. [14] at some points in time; however, differences exist to varying degrees (Figure 8). For example, the method of Murray et al. [14] clearly misclassified some water pixels as the tidal flats between 1987 and 1989, such that the extent of the tidal flats were significantly overestimated. Furthermore, between 1990 and 1992, their results were underestimated owing to the omission of some tidal flat pixels (Figure 9).

It has become a commonly used method to assess the classification accuracy by reference to high-resolution Google Earth images and time series Landsat observations in the GEE cloud platform (e.g., [14,17,50]). To further validate our approach, we adopted similar methods to Murray et al. [14] in evaluating the accuracy of our estimations. First, the power analysis method was used to estimate the validation sample size. The following equation can be used to calculate the required sample size *n* [51]:

(3) $$n=\frac{z_{\alpha /2}^{2}P\left(1-P\right)}{h^{2}}$$

where *z_α_*_/2_ is the critical value of the normal distribution for the two-tailed significance level α; *P* is an estimate of the actual population value, and; *h* is the half-width of the desired confidence interval. By convention, the significance level α was set at 0.05; thus, *z_α_*_/2_ is 1.96.

**Figure 10:**
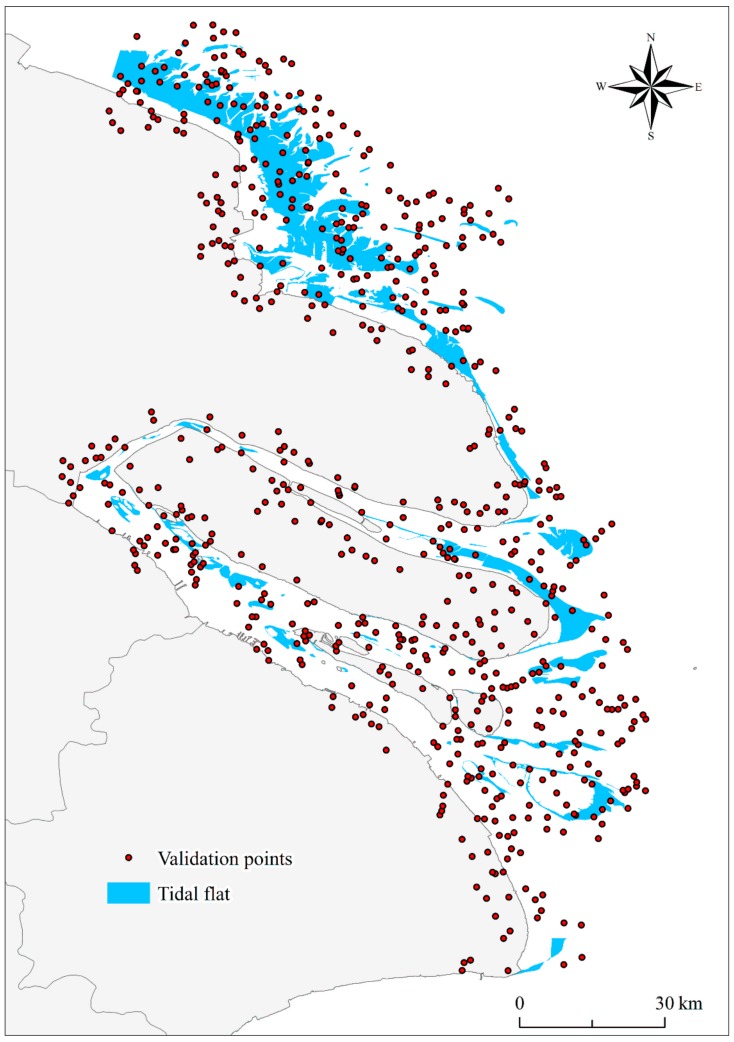
Distribution of the 664 validation sample points in 2018.

Here, we take our result of 2018 as an example for illustration. According to Equation (3), we calculated that 664 samples were sufficient to validate the accuracy of estimates. Thus, we generated 664 validation sets by stratified random sampling method (Figure 10). We then assigned the classes to each validation sample by referring to Landsat time-series images and high-resolution Google Earth images. Therefore, a confusion matrix can be calculated to obtain the overall accuracy, Kappa coefficient, user accuracy, and producer accuracy of each class (Table 1). The confusion matrix indicated that the overall accuracy was 98.2%, and the Kappa coefficient was 0.92. This suggests that our proposed algorithm exhibited a good capacity for classification. Second, a bootstrapping technique was used to estimate the confidence intervals of accuracies in a relatively unbiased manner [52]. Bootstrapping was performed with 1000 replications to generate 95% confidence intervals to assess the uncertainty surrounding the outcomes. We eventually arrived at a user accuracy interval of 84.1% to 96.6% for the tidal flat class, and the overall accuracy was 96.7% to 99.0%. We further derived that the total tidal flat area was between 924.0 km^2^ and 1233.7 km^2^ in 2018. Finally, we visually interpreted the tidal flat extent of the study area in 2018 and compared this with the extraction results in this study. The intersection of the extracted and interpreted areas was 96.2% of the interpreted area.

### 4.4. Potential Applications

In the present study, uncertainty in the results seems to be inevitable. The 16 days revisit period of Landsat images, combined with cloud cover issues and seasonal limitations, cannot resolve the rapidity of tidal variations. Consequently, the probability of capturing images at low tide is low [9]. Thus, uncertainty exists regarding the extent of the tidal flats derived from Landsat images at relatively low but varying tide levels. However, by taking the union of the extraction results from all available Landsat images of the study area over one year, we derived the areal extent of tidal flats at an extremely low tide level available by remote sensing for every location across the entire study area. By producing an annual dataset with a high temporal resolution, we greatly reduced the uncertainty, such that the long-term trend should be acceptable. In the future, as more remote sensing images with higher spatial and temporal resolution become available, e.g., Landsat 8 and Sentinel-2 satellite images, there will be greater opportunity for capturing images at extremely low tides. Therefore, our proposed method has the potential and scalability to be applied to other study areas in the world.

Our derived annual dataset of the tidal flat extent over the past 45 years will substantially contribute to understanding the past, present, and future of the tidal flats, the poorly known coastal ecosystems in the Yangtze River Delta. Furthermore, the dataset can be used to support a wide variety of models in coastal areas, such as the social-ecological system model [53], coastal landscape change model [54], morphological evolution model [55], coastal carbon cycle models [56], and coastal vulnerability model [49].

## 5. Conclusions

It is vital to understand the spatiotemporal evolution of tidal flats for regional sustainable development and global ecological security. In this study, we identified the 45-year variations of tidal flats from all available Landsat images in the Yangtze River Delta. To the best of our knowledge, this is the most comprehensive spatiotemporal tidal flat dataset in existence for this study area, covering the period 1974–2018. Our main findings are as follows: (1) Over the past 40 years, the Jiangsu coast and Yangtze estuary zone have, on average, accounted for 71.2% and 28.8%, respectively, of the tidal flat areas in the study area. The North Branch, Chongming Dongtan, South Branch, Changxing, Hengsha, Jiuduansha, and Pudong bank have accounted for 21.7%, 22.3%, 8.4%, 6.7%, 7.0%, 16.5%, and 17.3%, respectively, of the Yangtze estuary zone. (2) The areal extent of the tidal flats has generally declined in the study area since 1974. The tidal flat area reached a maximum in 1980. Since then, declines in the area have been conspicuous, with rates of decrease of 12.3 km^2^/year (*r*^2^ = 0.519) and 4.8 km^2^/year (*r*^2^ = 0.475), accounting for net losses of 40.6% and 38.8% for the Jiangsu coast and Yangtze estuary zone, respectively. (3) Reduced sediment load and land reclamation are the two main causes of the loss of tidal flat areas over the time scale of the study. The intensification of human activities and rising sea levels suggest that unless effective management measures are implemented, tidal flats and their associated ecosystem services will incur increasingly severe damage in the future. In addition, it should be noted that although this study was specific to the Yangtze River Delta, the proposed method has the potential to be used in other estuaries and coastal areas at a larger spatial scale. Our method is robust and scalable and is thus expected to achieve more accurate tidal flat data when more frequent remote sensing observations and efficient techniques of tidal flat extraction are developed.

## Figures and Tables

**Table 1:** The confusion matrix calculated from the 664 validation samples points for the tidal flat map in 2018.

		Reference			
		Tidal Flat	Other	Total	User Accuracy (%)
Classified	Tidal flat	82	6	88	93.2
	Other	6	570	576	99.0
	Total	88	576		
	Producer accuracy (%)	93.2	99.0		
	Overall accuracy (%)				98.2
	Kappa				0.92
